# Changes in bile acid profiles and short-term weight loss in response to a low-calorie diet: a pilot study

**DOI:** 10.3389/fnut.2025.1598933

**Published:** 2025-11-11

**Authors:** Ming-Xia Jiang, Chen-Jie Ling, Si-Cheng Qian, Jing-Nan Zhai, Jin-Yuan Huang, Ke-Ke Su, Xiang-Wei Wu, Qing-Zhen Han, Lin Wang

**Affiliations:** 1 Department of Clinical Nutrition, The Fourth Affiliated Hospital of Soochow University, Suzhou, China; 2 Clinical Testing Center, The Fourth Affiliated Hospital of Soochow University, Suzhou, China

**Keywords:** bile acids, gut microbiota, low-calorie diet, weight loss, obesity

## Abstract

**Background:**

Dietary intervention-induced modulation of bile acids may enhance metabolic homeostasis and facilitate weight reduction. We aimed to elucidate the role of bile acids and their association with the gut microbiota during weight reduction induced by a low-calorie diet (LCD).

**Methods:**

Twelve participants were enrolled, and body composition, serum bile acid profiles, and gut microbiota were analyzed at baseline and after a 4-week LCD intervention.

**Results:**

The LCD significantly reduced body weight (3.39 ± 1.99 kg, *p* < 0.001) and body fat (2.47 ± 2.00 kg, *p* = 0.001), accompanied by decreases in skeletal muscle mass (0.59 ± 0.55 kg, *p* = 0.003), serum iron, magnesium, uric acid, and triglyceride (TG) levels. Notably, serum lithocholic acid levels increased significantly and were negatively correlated with TG levels and positively correlated with *Faecalibacterium* abundance. The LCD also decreased the relative abundances of *Streptococcaceae* and *Streptococcus*, while increasing *Porphyromonadaceae*, *Christensenellaceae*, *Parabacteroides*, and *Oscillospira*.

**Conclusion:**

These findings suggest that increased LCA is associated with metabolic improvements during LCD intervention.

## 1 Introduction

Obesity has become a serious public health concern worldwide, including in China (1). Among the various treatment strategies, dietary modification remains a cornerstone of weight loss management due to its safety and effectiveness. However, the success of dietary interventions largely depends on dietary adherence, which poses a significant challenge, especially with low-calorie diets (LCDs), one of the most widely used approaches. LCDs have been shown to improve dyslipidemia, insulin resistance, and hypertension, thereby promoting overall metabolic health (2, 3). Elucidating the mechanisms through which LCDs regulate metabolism and induce weight reduction is essential for optimizing dietary strategies and improving obesity management.

It is widely recognized that dietary interventions exert their effects, in part, by modulating the gut microbiota (4). Bile acids (BAs), which are metabolized by gut microbiota, act as signaling molecules that regulate key host metabolic pathways. These effects are primarily mediated through the nuclear farnesoid X receptor (FXR) and the G protein-coupled membrane receptor 5 (TGR5), which coordinate the crosstalk between the gut microbiota and host metabolism (5). Emerging evidence indicates that BAs influence both energy intake and expenditure pathways, thereby contributing to weight loss. Peripherally, BA-activated TGR5 signaling induces the browning of white adipose tissue and enhances thermogenesis in brown adipose tissue (6), promoting energy consumption and fat catabolism. Centrally, activation of the BA-TGR5 axis reduces appetite by modulating hypothalamic neurons involved in hunger and satiety, while also stimulating sympathetic nervous system activity to increase energy expenditure (7, 8). Additionally, BA-mediated TGR5 activation suppresses inflammation and promotes glucagon-like peptide-1 (GLP-1) secretion, further supporting metabolic benefits (6). These pleiotropic actions position BAs as key mediators in the regulation of body weight (WB) and metabolism.

Accumulating evidence suggests that dietary interventions alleviate obesity and improve obesity-related metabolic disorders by modulating BA metabolism. Time-restricted feeding has been shown to restore impaired ileal BA signaling in diet-induced obese mice, accompanied by improved metabolic outcomes (9). Similarly, diet-induced weight loss reverses obesity-associated alterations in BA profiles in animal models (10). Ketogenic diets influence serum BA composition, leading to reduced WB and fasting glucose levels by limiting calorie absorption (11). Notably, reduced circulating levels of specific BAs may contribute to rebound weight gain following calorie restriction (12). These findings highlight the pivotal role of BAs in determining the efficacy of dietary interventions. Although LCDs have been reported to alter serum BA profiles and modulate BA metabolism (13, 14), the relationship between these changes and LCD-induced weight loss remains poorly understood, and human data are limited.

In this study, we investigated serum BA profiles and gut microbiota composition before and after an LCD intervention. Our objective was to clarify the role of BAs and their interactions with gut microbiota in mediating LCD-associated weight loss.

## 2 Methods and methods

### 2.1 Participants and ethical approval

**Figure 1:**
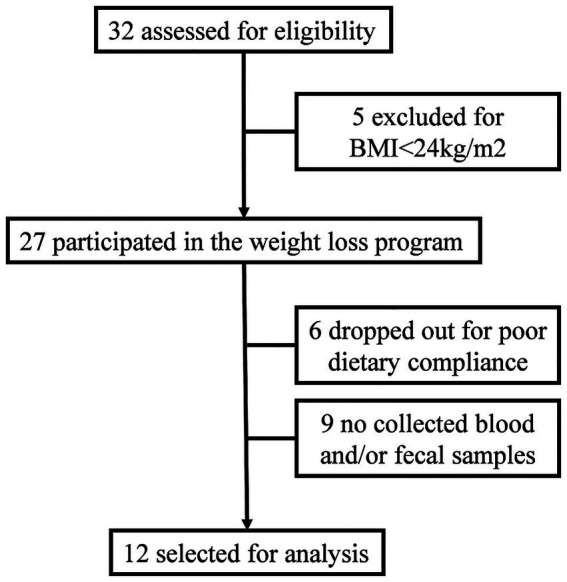
Participant flow diagram.

Participants were recruited from among the medical staff who voluntarily enrolled in a weight-loss management program at the Clinical Nutrition Department of the Fourth Affiliated Hospital of Soochow University. Recruitment information was released by the network within the hospital. The participant selection process was shown in Figure 1. Inclusion criteria were a body mass index (BMI) greater than 24 kg/m^2^ and age between 18 and 65 years. Exclusion criteria included the presence of severe medical conditions (e.g., cardiovascular disease, liver or kidney dysfunction, gastrointestinal disorders, malignant tumors), pregnancy or lactation, use of antibiotics or probiotics within the past 3 months, or unwillingness to comply with the program. This study was approved by the Ethics Committee of the Fourth Affiliated Hospital of Soochow University (ethics review number: 240029), and all participants provided written informed consent.

### 2.2 Intervention

All participants followed an LCD with a daily intake of 1,200 kcal for men and 1,000 kcal for women. The energy supply distribution of macronutrients was 25% protein, 30% fat, and 45% carbohydrates. Face-to-face dietary counseling and a nutrient-quantified recipe were provided to the participants by dietitians. Participants were instructed to weigh each meal and upload photographs to monitor adherence. Those who failed to have meals as instructed for more than 3 days a week were dropped out. The intervention period lasted 4 weeks.

### 2.3 Body composition assessment

Body composition parameters including WB, body fat (BF), skeletal muscle mass (SMM), percentage of body fat (PBF), and visceral fat area (VFA), were measured 1 day before and after the intervention using inBody S10 (BiospaceCo., Ltd., Seoul, Korea). All measurements were performed in the morning following an overnight fast.

### 2.4 Serum biochemical index assessment

Venous blood samples were collected from the antecubital vein after an 8-h overnight fast, 1 day before and after the LCD. Serum samples were obtained by centrifugation at 2,500 rpm for 10 min. Biochemical indexes, including aspartate transaminase (AST), alanine transaminase (ALT), prealbumin, urea, creatinine, uric acid, calcium (Ca), phosphorus (P), magnesium (Mg), iron (Fe), glucose, triglycerides (TG), total cholesterol (TC), low-density lipoprotein (LDL), and high-density lipoprotein (HDL), were measured using an Mindray automated biochemical analyzer (Shenzhen Mindray Bio-Medical Electronics Co., LTD, Shenzhen, China) at the Clinical Testing Center of the hospital.

### 2.5 Serum bile acid quantification

Serum bile acid profiles were quantified using liquid chromatography–tandem mass spectrometry (LC–MS/MS). A total of 100 μL of serum samples were vortexed with 600 μL of methanol for 60 s. The mixture was then centrifuged at 12,000 rpm and 4 °C for 10 min. Following centrifugation, 400 μL of the supernatants were evaporated to dryness in a vacuum concentrator (Eppendorf AG, Hamburg, Germany) and reconstituted in 100 μL of 30% methanol. In addition, the bile acid standard was weighed and prepared in methanol as a 1,000 μg/mL stock solution, followed by serial dilution with 30% methanol to create a 10-point calibration curve. Samples were analyzed using an LC–MS/MS system (ABSciex Co., Ltd., Boston, Massachusetts, United States) equipped with an ACQUITY UPLC BEH C18 column (Waters Co., Boston, Massachusetts, United States). Chromatographic separation was achieved with a column temperature of 40 °C and an injection volume of 5 μL. The mobile phase consisted of (A) 0.01% formic acid in water and (B) acetonitrile, delivered at a flow rate of 0.25 mL/min with the following gradient: 0–4 min, 25% B; 4–9 min, 25–30% B; 9–14 min, 30–36% B; 14–18 min, 36–38% B; 18–24 min, 38–50% B; 24–32 min, 50–75% B; 32–33 min, 75–90% B; 33–35.5 min, 90–25% B. Mass spectrometric analysis was conducted in negative electrospray ionization (ESI) mode using multiple reaction monitoring (MRM). The ion source parameters were as follows: source temperature, 500 °C; spray voltage, −4,500 V; curtain gas, 30 psi; collision gas, 6 psi; nebulizing and auxiliary gas, 50 psi. Data acquisition and quantification were performed using Analyst software. The following BAs were measured: lithocholic acid (LCA), allolithocholic acid (alloLCA), isolithocholic acid (isoLCA), 23-nordeoxycholic acid (NorDCA), 7-ketolithocholic acid (7-ketoLCA); 12-ketolithocholic acid (12-ketoLCA), 3*β*-ursodeoxycholic acid (β-UDCA), deoxycholic acid (DCA), tauroursodeoxycholic acid (TDCA), chenodeoxycholic acid (CDCA), taurochenodeoxycholic acid (TCDCA), ursodeoxycholic acid (UDCA), cholic acid (CA), norcholic acid (NorCA), allocholic acid (ACA), 3β-cholic acid (*β*-CA), glycolithocholic acid (GLCA), glycochenodeoxycholic acid (GCDCA), glycoursodeoxycholic acid (GUDCA), glycodeoxycholic acid (GDCA), glycocholic acid (GCA), taurocholic acid (TCA), tauro-*α*-muricholic acid (T-α-MCA), taurohyocholic acid (THCA), and chenodeoxycholic acid 24-Acyl-β-D-glucuronide (CDCA-G). The averages of recovery rate, within-day precision, and between-day precision were 94.7, 4.8, and 7.7%, respectively.

### 2.6 16S rRNA sequencing analysis

Fecal samples were obtained before and after the LCD intervention. Samples were lyophilized, crushed, and lysed. Following centrifugation, DNA was extracted using a commercial kit (ZEPING Biotech Co., Ltd., Beijing, China). The V3-V4 region of the 16S rRNA gene was amplified via polymerase chain reaction (PCR). Sequencing libraries were prepared from the PCR products and sequenced using the MiSeq platform (Illumina Inc., San Diego, CA, United States). Amplicon sequence variants (ASVs) were analyzed using QIIME2. Briefly, after filtering the raw reads by removing the primers, the paired-end reads were merged. The merged data was denoised using DADA2 to generate ASVs. Frequency-based filtering was applied to remove singletons, and chimera removal was performed to eliminate chimeric ASVs. Alpha diversity indices (Observed species, Chao1, Simpson index, and Shannon index) were compared using the Kruskal-Wallis test. Beta diversity was assessed using principal coordinate analysis (PCoA) based on four distinct distance metrics: the Jaccard index, Bray–Curtis dissimilarity, and both unweighted and weighted UniFrac distances. Linear discriminant analysis effect size (LEfSe) analysis was used to identify differentially abundant taxa.

### 2.7 Statistical analysis

Statistical analyses were performed using SPSS (version 26) and R Software (version 4.3.3). Changes in body composition and serum biochemical indices before and after LCD were evaluated using the paired Student’s *t*-test. Changes in BA levels were analyzed using either paired Student’s *t*-test or Wilcoxon rank-test, as appropriate. Correlation analyses were performed using Kendall’s Tau-b, and only correlation coefficients>0.6 were reported. *Δ*-values (computed as post- minus pre-intervention) were employed in the correlation analysis. A *p*-value < 0.05 was considered statistically significant.

## 3 Results

Our study included 12 overweight or obese participants in the analysis. Participant characteristics and body composition before and after the LCD were summarized in Table 1. The mean age of the participants was 29.2 ± 4.04 years, with 75% being women. Post-LCD, the average weight loss was 3.39 ± 1.99 kg (*p* < 0.001), corresponding to a 4.58% reduction from baseline weight. The mean decrease in BMI was 1.28 ± 0.75 kg/m^2^ (*p* = 0.002). Significant reductions were also observed in BF (2.47 ± 2.00 kg, *p* = 0.001), PBF (1.83 ± 2.18%, *p* = 0.014), SMM (0.59 ± 0.55 kg, *p* = 0.003), and VFA (11.49 ± 8.78 cm^2^, *p* = 0.001).

**Table 1:** General information and body composition of participants pre- and post-LCD.

Variable	Pre-LCD	Post-LCD	Difference value	*p-*value
N	12	12	–	–
Sex (f/m)	9/3	–	–	–
Age (y)	29.2 ± 4.04	–	–	–
Body weight (kg)	73.28 ± 7.37	69.89 ± 6.94	−3.39 ± 1.99	**<0.001**
Percentage of weight loss (%)	–	–	−4.58 ± 2.55	**<0.001**
BMI (kg/m^2^)	27.21 ± 2.38	25.93 ± 2.21	−1.28 ± 0.75	**0.002**
Body fat (kg)	25.56 ± 3.72	23.09 ± 4.05	−2.47 ± 2.00	**0.001**
Percentage of body fat (%)	35.06 ± 5.11	33.23 ± 5.91	−1.83 ± 2.18	**0.014**
Skeletal muscle mass (kg)	26.5 ± 4.68	25.91 ± 4.70	−0.59 ± 0.55	**0.003**
Visceral fat area (cm^2^)	115.13 ± 25.3	103.64 ± 24.85	−11.49 ± 8.74	**0.001**

Bold values denote statistical significance (*p* < 0.05).

Serum biochemical indices before and after the LCD intervention were presented in Table 2. The LCD significantly reduced prealbumin, Mg, Fe, uric acid, and TG levels, with mean decreases of 31.25 ± 37.5 mg/L (*p* = 0.015), 0.04 ± 0.05 mmol/L (*p* = 0.016), 4.13 ± 5.33 μmol/L (*p* = 0.021), 35.12 ± 53.01 μmol/L (*p* = 0.042) and 0.38 ± 0.71 mmol/L (*p* = 0.038), respectively.

**Table 2:** Serum biochemical indexes of participants pre- and post-LCD.

Variable	Pre-LCD	Post-LCD	Difference value	*P-*value
AST (U/L)	21.28 ± 10.94	23.86 ± 15.83	2.58 ± 6.31	0.239
ALT (U/L)	31.16 ± 32.98	33.23 ± 40.57	2.08 ± 9.71	0.937
Prealbumin (mg/L)	254.5 ± 47.49	223.25 ± 44.71	−31.25 ± 37.5	**0.015**
Urea (mmol/L)	4.91 ± 0.96	4.75 ± 1.39	−0.17 ± 1.33	0.668
Creatinine (μmol/L)	64.33 ± 16.07	64.59 ± 14.13	0.27 ± 4.5	0.841
Uric acid (μmol/L)	315.82 ± 90.58	280.7 ± 80.13	−35.12 ± 53.01	**0.042**
Ca (mmol/L)	2.38 ± 0.09	2.35 ± 0.08	−0.03 ± 0.09	0.303
P (mmol/L)	1.21 ± 0.12	1.21 ± 0.19	0 ± 0.17	0.934
Mg (mmol/L)	0.89 ± 0.06	0.84 ± 0.05	−0.04 ± 0.05	**0.016**
Fe (μmol/L)	15.92 ± 8.33	11.79 ± 6.12	−4.13 ± 5.33	**0.021**
Glucose (mmol/L)	5.1 ± 0.72	5.15 ± 0.51	0.04 ± 0.54	0.58
TG (mmol/L)	1.94 ± 1.15	1.23 ± 0.42	−0.71 ± 1.05	**0.038**
TC (mmol/L)	4.7 ± 0.55	4.32 ± 0.73	−0.38 ± 0.71	0.088
HDL (mmol/L)	1.24 ± 0.17	1.3 ± 0.29	0.06 ± 0.23	0.563
LDL (mmol/L)	3.02 ± 0.45	2.72 ± 0.72	−0.3 ± 0.64	0.14

Bold values denote statistical significance (*p* < 0.05). AST, aspartate transaminase; ALT, alanine transaminase; Ca, calcium; P, phosphorus; Mg, magnesium; Fe, iron; TG, triglycerides; TC, total cholesterol; LDL, low-density lipoprotein; HDL, high-density lipoprotein.

**Figure 2:**
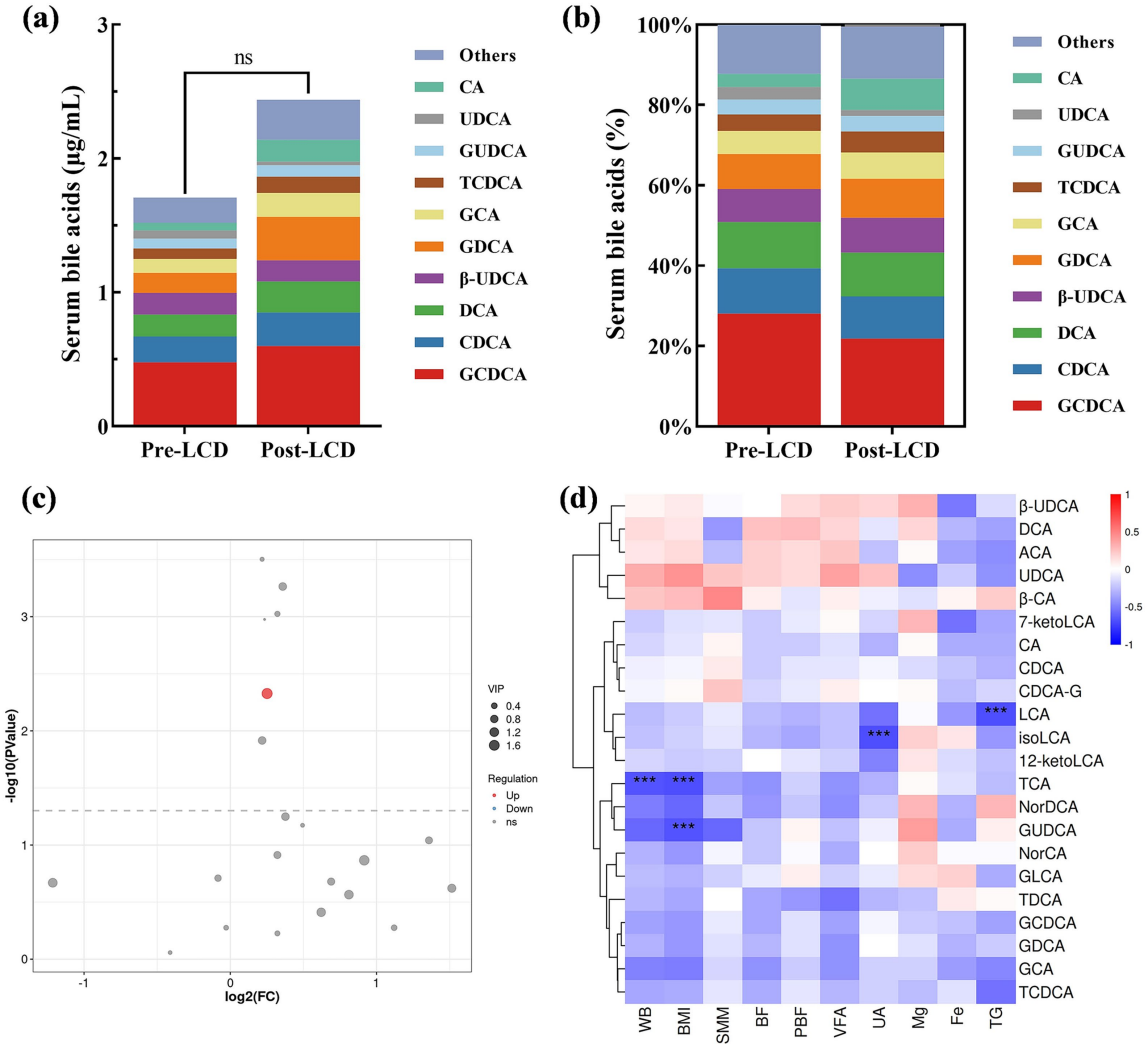
Alterations in serum BAs after the LCD intervention. **(a)** Total serum BA levels and **(b)** BA composition. **(c)** Volcano plot analysis, and red dots indicate elevated BAs post-LCD. **(d)** Correlation analysis of serum BA level variations with body composition parameters and biochemical indices. Only correlation coefficients >0.6 were shown. Red: positive correlation; blue: negative correlation. Darker shades indicate stronger correlations. ***p < 0.001.

Although total serum BA levels showed an increase in post-LCD, this change did not reach statistical significance (Figure 2a). Changes in BA composition were illustrated in Figure 2b and detailed in Table 3. A volcano plot identified a significant increase only in LCA levels post-LCD, based on screening thresholds of *p* < 0.05 and fold change >1 (Figure 2c). To investigate the relationship between BA changes and weight loss, correlation analyses were performed. Negative correlations were observed between changes in the following pairs: TCA and BW (*r* = −0.66, *p* < 0.001), TCA and BMI (*r* = −0.68, *p* < 0.001), GUDCA and BMI (*r* = −0.66, *p* < 0.001), isoLCA and UA (*r* = −0.69, *p* < 0.001), and LCA and TG (*r* = −0.68, *p* < 0.001) (Figure 2d).

**Table 3:** Serum BA levels of participants pre- and post-LCD (ng/ml).

Variable	Pre-LCD	Post-LCD	Difference value	*P*-value
LCA	24.44 ± 6.25	29.15 ± 7.79	4.71 ± 5.52	**0.013**
alloLCA	5.83 ± 0.59	6.28 ± 0.47	0.45 ± 0.59	0.069
TDCA	33.16 ± 27.64	84.47 ± 140.52	51.31 ± 129.6	0.091
CA	56.56 ± 52.41	163.7 ± 210.89	107.14 ± 211.67	0.107
DCA	163.45 ± 137.6	230.6 ± 163.14	67.15 ± 133.83	0.11
TCA	19.72 ± 22.16	37.34 ± 49.46	17.62 ± 45.02	0.136
THCA	1.26 ± 0.67	3.16 ± 3.21	1.81 ± 3.57	0.15
GCA	102.82 ± 76.93	178.97 ± 183.37	76.16 ± 175.36	0.161
7-ketoLCA	3.65 ± 3.93	5.9 ± 5.28	2.25 ± 5.48	0.183
UDCA	72.04 ± 104.38	35.93 ± 52.71	−39.37 ± 83.71	0.196
GDCA	150.16 ± 145.81	325.28 ± 507.85	175.12 ± 457.39	0.212
TCDCA	79.37 ± 92.64	121.35 ± 148.53	41.98 ± 133.77	0.3
isoLCA	9.49 ± 4.42	8.33 ± 2.5	−1.16 ± 3.55	0.305
GLCA	12.21 ± 4.77	15.34 ± 11.08	3.13 ± 10.59	0.351
NorCA	1.88 ± 1.1	2.43 ± 1.45	0.54 ± 1.95	0.355
ACA	16.67 ± 38.19	12.52 ± 22.39	−4.15 ± 16.66	0.406
12-ketoLCA	5.78 ± 4	6.83 ± 4.26	1.06 ± 4.27	0.432
GUDCA	72.14 ± 69.34	84.28 ± 87.57	12.14 ± 51.6	0.432
NorDCA	1.39 ± 0.85	1.76 ± 1.25	0.31 ± 1.21	0.433
CDCA	193.31 ± 149.5	249.71 ± 225.24	56.4 ± 241.18	0.435
GCDCA	477.53 ± 319.64	599.81 ± 665.9	122.28 ± 600.18	0.495
CDCA-G	18.21 ± 9.64	17.2 ± 7.17	−1.01 ± 8.71	0.695
T-α-MCA	11.8 ± 9.32	45.66 ± 99.61	50.85 ± 127.2	0.779
β-CA	3.34 ± 2.83	4.28 ± 5.09	0.95 ± 5.99	0.959
β-UDCA	161.52 ± 278.24	158.54 ± 325.49	−2.99 ± 267.18	0.97

LCA, lithocholic acid; alloLCA, allolithocholic acid; TDCA, tauroursodeoxycholic acid; CA, cholic acid; DCA, deoxycholic acid; TCA, taurocholic acid; THCA, taurohyocholic acid; GCA, glycocholic acid; 7-ketoLCA, glycocholic acid; UDCA, ursodeoxycholic acid; GDCA, glycodeoxycholic acid; TCDCA, taurochenodeoxycholic acid; isoLCA, isolithocholic acid; GLCA, glycolithocholic acid; NorCA, norcholic acid; ACA, allocholic acid; 12-ketoLCA, 12-ketolithocholic acid; GUDCA, glycoursodeoxycholic acid; NorDCA, 23-nordeoxycholic acid; CDCA, chenodeoxycholic acid; GCDCA, glycochenodeoxycholic acid; CDCA-G, chenodeoxycholic acid 24-Acyl-β-D-glucuronide; T-α-MCA, tauro-α-muricholic acid; β-CA, 3β-cholic acid; β-UDCA, 3β-ursodeoxycholic acid. Bold values denote statistical significance (*p* < 0.05).

**Figure 3:**
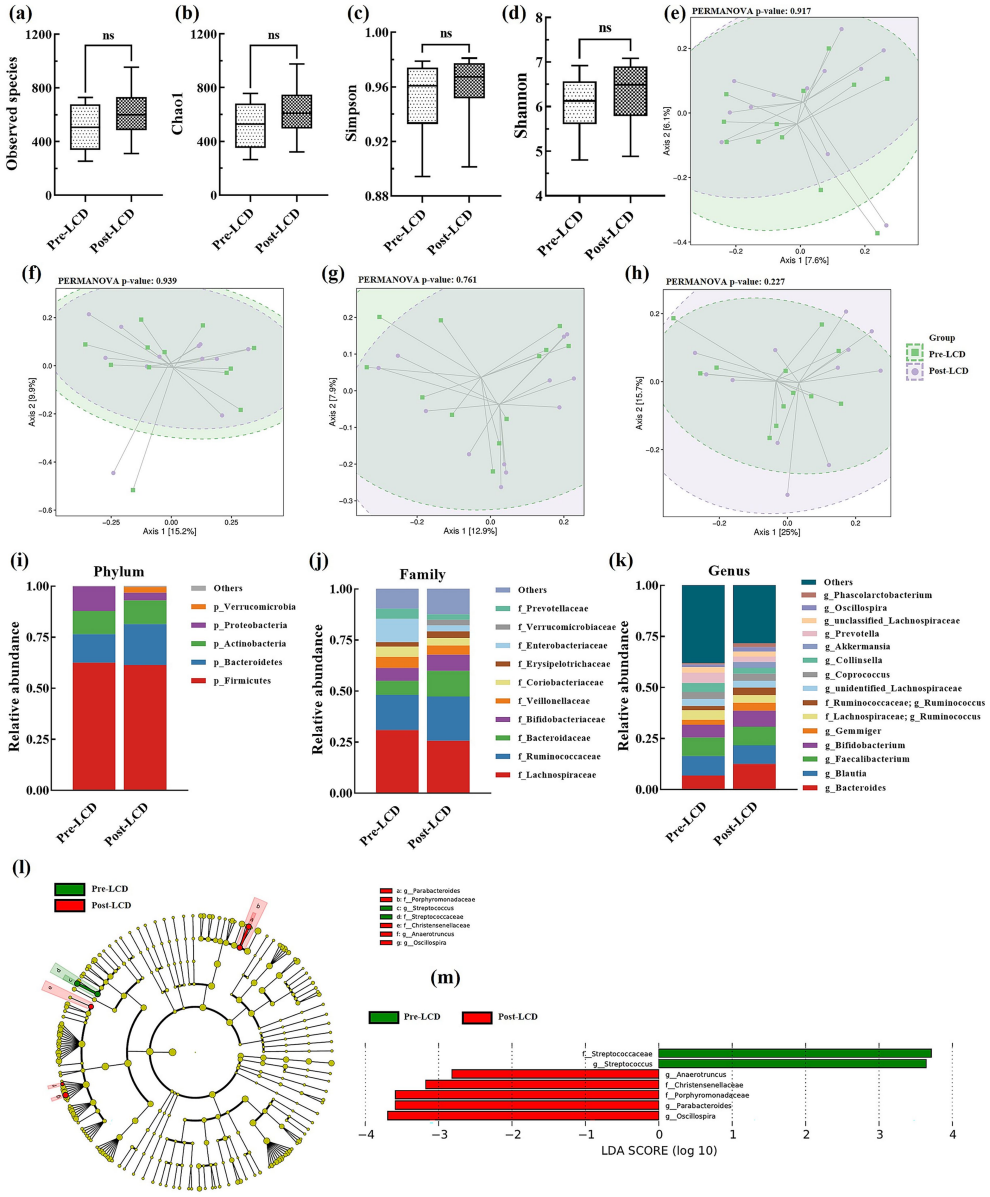
Alterations in gut microbiota after LCD intervention. Alpha diversity includes **(a)** Observed species, **(b)** Chao 1, **(c)** Simpson index, and **(d)** Shannon index; PCoA plots: **(e)** Jaccard, **(f)** Bray-Curtis, **(g)** Unweighted UniFrac, and **(h)** Weighted UniFrac; Gut microbiota composition at the **(i)** phylum, **(j)** family, and **(k)** genus levels; **(l)** LEfSE analysis and **(m)** LDA scores. LEfSe, linear discriminant analysis effect size; LDA, linear discriminant analysis.

Gut microbiota, known to influence BA metabolism, was analyzed from stool samples collected before and after the LCD. No significant differences were detected in alpha-diversity indices, including observed species, Chao1, Simpson, and Shannon indexes (Figures 3a–d). At the ASV level, minimal overall compositional changes in gut microbiota were observed post-LCD (Figures 3e–h). Microbial community compositions at the phylum, family, and genus levels are presented in Figures 3i–k, respectively. LEfSe analysis identified significantly altered taxa following LCD intervention. At the family level, the relative abundance of *Streptococcaceae* significantly decreased, whereas *Porphyromonadaceae* and *Christensenellaceae* increased. At the genus level, *Streptococcus* abundance was significantly reduced, while *Parabacteroides*, *Oscillospira*, and *Anaerotruncus* were significantly increased (Figures 3l,m).

**Figure 4:**
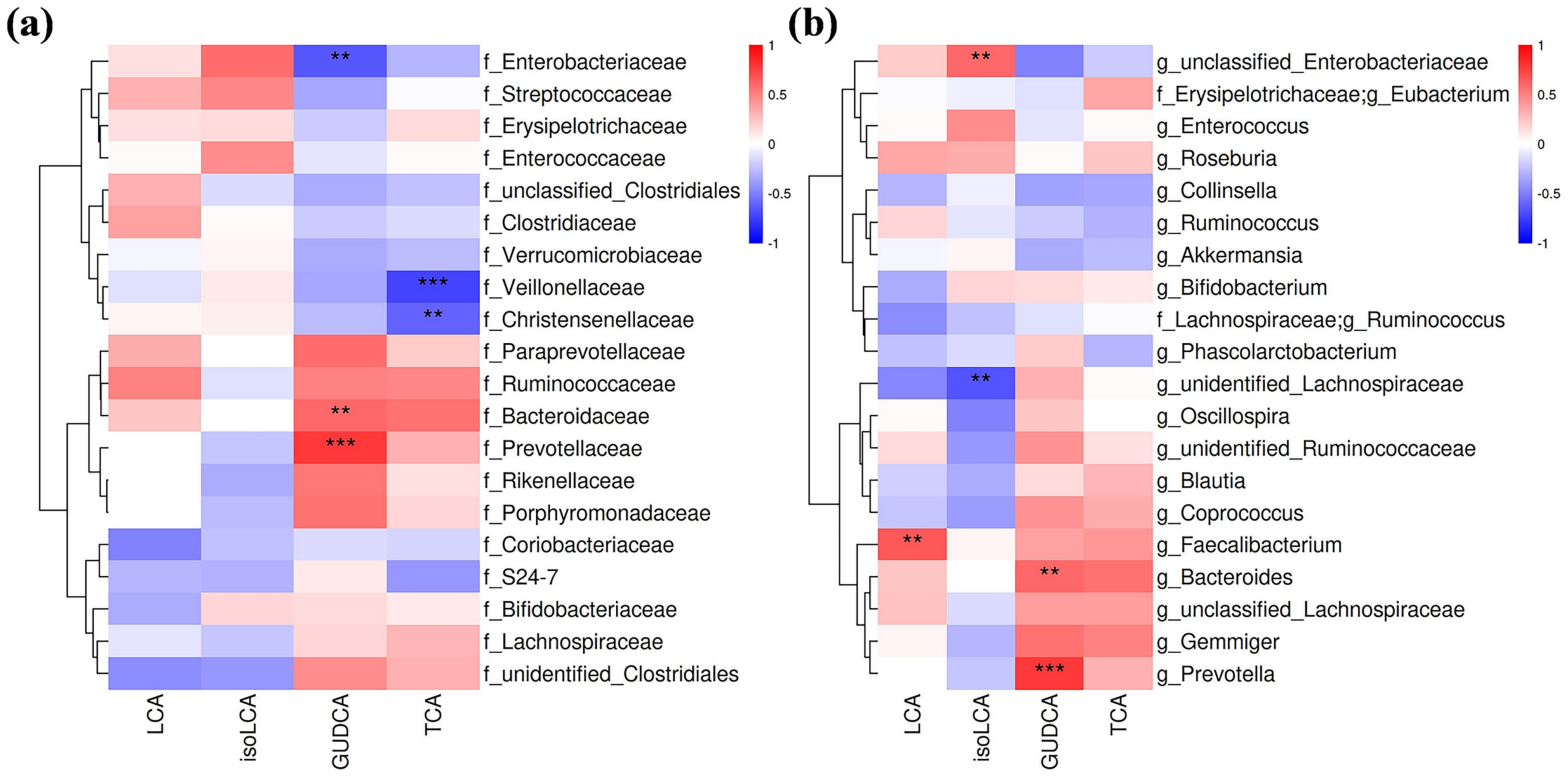
Correlation analysis of serum BA level changes and alterations in gut microbiota at the family **(a)** and genus levels **(b)**. Only correlation coefficients >0.6 were shown. Red: positive correlation; blue: negative correlation. Darker shades indicate stronger correlations; **p < 0.01; ***p < 0.001.

Associations between changes in gut microbiota and specific BAs—including LCA, iso-LCA, TCA, and GUDCA—were analyzed. Notably, changes in LCA levels correlated positively with the genus *Faecalibacterium* (*r* = 0.60, *p* = 0.005). GUDCA changes showed positive correlations with families *Bacteroidaceae* (*r* = 0.67, *p* = 0.001) and *Prevotellaceae* (*r* = 0.8, *p* < 0.001) and their constituent genera *Bacteroides* (*r* = 0.60, *p* = 0.005) and *Prevotella* (*r* = 0.80, *p* < 0.001), but negative correlations with the family *Enterobacteriaceae* (*r* = −0.64, *p* = 0.003). TCA changes were inversely correlated with families *Veillonellaceae* (*r* = −0.72, *p* < 0.001) and *Christensenellaceae* (*r* = −0.61, *p* = 0.004) (Figures 4a,b).

## 4 Discussion

Gut microbiota-BA crosstalk contributes to weight reduction during dietary interventions. In this study, we analyzed changes in the serum BA profile and gut microbiota composition following a short-term LCD intervention. The LCD significantly reduced BW and BF, alongside decreases in serum Fe, Mg, uric acid, and TG. Both the serum BA profile and gut microbiota composition shifted after the LCD intervention.

BAs play a critical role in regulating energy expenditure and glucolipid metabolism and are recognized as key mediators of anti-obesity effects. Previous studies report that 3- and 6-month LCD interventions reduce serum BA levels in overweight and obese adults (15, 16). In contrast, our 4-week LCD intervention did not observe a significant reduction in total serum BAs. This discrepancy may be attributed to differences in intervention duration and the magnitude of weight loss. Heianza et al. demonstrate that larger decreases in serum BA levels correlate with greater weight loss (15). Further analysis of BA composition revealed a significant increase in serum LCA following LCD, which correlated with TG reduction. Prior studies indicate that individuals with high BMI tend to have lower LCA proportions (17, 18), and weight loss interventions can elevate LCA levels (19), consistent with our findings. Additionally, LCA administration has been shown to alleviate high-fat diet-induced obesity and hyperlipidemia (18, 19), and serum LCA levels are negatively associated with TG levels (20). Our study also found negative correlations between weight loss and changes in GUDCA and TCA; however, increases in these BAs did not reach statistical significance after LCD. Previous research suggests that GUDCA administration improves obesity and glucolipid metabolism (21), while TCA stimulates secretion of glucagon-like peptide-1(GLP-1) and peptide YY (PYY), enhances satiety, and improves insulin sensitivity (22–24). These findings suggest that alterations in BA composition may partially mediate LCD-induced weight loss.

Despite no significant changes in *α*- and *β*-diversity, specific gut microbiota exhibited compositional shifts following the 4-week LCD intervention. At the genus level, relative abundances of *Streptococcus* decreased, while *Parabacteroides*, *Oscillospira*, and *Anaerotruncus* increased. A meta-analysis has shown that *Streptococcus* is more abundant in adults with obesity compared to those without (25), and weight loss following a low-carbohydrate diet reduces its relative abundance (26). However, Li et al. report a negative association between BMI and *Streptococcus* abundance, primarily driven by *Streptococcus thermophilus*, which is positively correlated with yogurt consumption (27). In individuals with obesity, *Streptococcus* abundance has been found to increase following probiotic and prebiotic interventions (28, 29), but decrease after flavonoid supplementation (30). These fluctuations may reflect species-specific differences within the genus, which may be affected by different intervention methods, highlighting the importance of analyzing *Streptococcus* at the species level rather than the genus level. Short-chain fatty acids (SCFAs) have been shown to promote intestinal health and mitigate obesity. The mechanisms involve promoting satiety hormone production from enteroendocrine L-cells, enhancing fat oxidation, and resting energy expenditure (31). *Parabacteroides* and *Oscillospira* are both SCFA-producing bacteria; notably, *Parabacteroides distasonis* and *Parabacteroides goldsteinii* have been demonstrated to alleviate obesity (32), and *Oscillospira* is strongly negatively associated with obesity and metabolic diseases (33). Our results showed increased abundances of these two genera post-LCD, consistent with their putative beneficial roles. Conversely, *Anaerotruncus* has been positively correlated with obesity (34) and has been shown to decrease following a 9-week anti-obesity intervention (35). It has also been implicated in dietary cholesterol-induced hepatocellular carcinoma (36). While *Anaerotruncus* is generally considered detrimental to health, our study observed an unexpected increase in its relative abundance after LCD. To our knowledge, the effects of LCD on *Anaerotruncus* have not been previously reported. Given the limited sample size of this pilot study, this preliminary observation warrants further validation in larger, longitudinal studies. *Christensenellaceae* are widely regarded as beneficial bacteria linked to weight loss (37). Consistent with this, we observed an increase in the relative abundance of *Christensenellacea* post-LCD, corroborating findings from other weight-loss studies (38, 39).

Correlation analyses were conducted to explore BA-microbiota interactions. Changes in LCA were positively correlated with alterations in the genus *Faecalibacterium*. Jie et al. report that administration of *Faecalibacterium longum* increases serum LCA levels by promoting the relative abundance of LCA-producing bacteria and upregulating the 7α-dehydroxylation pathway. Increased LCA activates the FXR in the liver, downregulating lipid biosynthesis genes and upregulating lipid catabolism genes (20). Qu et al. suggest that increased serum LCA levels after calorie restriction activate AMP-activated protein kinase (AMPK) (40), which is a metabolic energy sensor and is lowly activated in obesity, insulin resistance, and chronic diseases (41). These indicate that strategies to increase LCA or *Faecalibacterium* abundances by regulating dietary components may contribute to weight loss using an LCD. Changes in GUDCA were positively correlated with shifts in the families *Bacteroidaceae* and *Prevotellaceae*, and the genera *Bacteroides* and *Prevotella*, but negatively correlated with changes in the family *Enterobacteriaceae*. We did not find other studies reporting the relationship between GUDCA and these bacteria. Chen et al. demonstrate that GUDCA decreases in patients with type 2 diabetes, and GUDCA intervention increases *Bacteroides vulgatus* and improves glucolipid metabolism (42). Alterations in TCA were negatively correlated with changes in families *Christensenellaceae* and *Veillonellaceae*. Genomic and metabolomic profiling analyses have shown that *Christensenella* species modify BAs via hydrolysis, dehydrogenation, amidation, and acylation (37). Furthermore, a negative correlation between *Veillonella* and stool TCA level has been observed in patients with nonalcoholic steatohepatitis (43). However, these bacterial changes did not reach statistical significance after the LCD intervention.

It is well established that calorie restriction leading to weight loss is often accompanied by skeletal muscle loss, which can be detrimental to overall health. In our study, we observed an average reduction of 0.59 kg in SMM, representing approximately 17.4% of total weight loss. Current evidence suggests that increased protein intake combined with strengthening exercises, such as resistance training or a combination of resistance and aerobic exercise, helps preserve muscle mass (44). In our study, protein intake ranged from 0.8 to 1.0 g/kg BW per day, and there was no mandatory exercise regimen; consequently, muscle loss occurred due to the absence of interventions aimed at muscle preservation. BAs are known to influence muscle health. Although we did not detect a direct correlation between BA levels and muscle loss, the observed elevation of LCA post-LCD has been suggested to promote skeletal muscle regeneration via the TGR5 receptor (45).

This study has several limitations. First, the small sample size resulted in low statistical power to detect significant findings, particularly in the analyses of BAs and the gut microbiota, which limits the reliability and generalizability of our results. Additionally, correlations involving BAs that did not change significantly are exploratory and should not be interpreted as evidence of an intervention effect. Second, the majority of participants in this study were women, and the study design lacked a control group. Moreover, the gut microbiota analysis utilized 16S rRNA sequencing, which does not allow for detection at the species-level taxonomic resolution.

In conclusion, a 4-week LCD intervention altered the serum BA profile and gut microbiota composition. Notably, LCD increased the relative abundance of *Porphyromonadaceae*, *Christensenellaceae*, *Parabacteroides*, *Oscillospira*, and *Anaerotruncus*, while decreasing *Streptococcaceae* and *Streptococcus*. Serum LCA levels were significantly elevated post-intervention, with LCA positively correlated with *Faecalibacterium* and negatively associated with serum TG levels. Considering the small sample size, these results needed to be confirmed by research with a larger sample size. Future studies will also include comparisons of different LCD interventions and functional studies to provide more comprehensive insights.

## Data Availability

The data presented in the study are deposited in the NCBI SRA repository, accession number PRJNA1353849.
